# Anti-Interferon Autoantibodies in Adult-Onset Immunodeficiency Syndrome and Severe COVID-19 Infection

**DOI:** 10.3389/fimmu.2021.788368

**Published:** 2021-12-22

**Authors:** Long-Fang Chen, Cheng-De Yang, Xiao-Bing Cheng

**Affiliations:** Department of Rheumatology and Immunology, Ruijin Hospital, Shanghai Jiao Tong University School of Medicine, Shanghai, China

**Keywords:** adult-onset immunodeficiency syndrome, coronavirus disease 2019 pneumonia, autoantibodies against IFNs, autoimmune disease, infectious disease

## Abstract

Adult-onset immunodeficiency syndrome due to anti-interferon (IFN)-γ autoantibodies has attracted much attention in recent years. It usually occurs in previously healthy people and usually presents as chronic, recurrent, and hard-to-control infections that can be effectively treated with aggressive antibiotic therapy. Adult-onset immunodeficiency syndrome is also referred to as AIDS-like syndrome. Anti-type I IFN (IFN-I) autoantibodies have been reported to play a significant role in the pathogenesis of coronavirus disease 2019 (COVID-19) and preexisting anti-IFN-I autoantibodies are associated with an increased risk of severe COVID-19. This review summarizes the effects of anti-IFN autoantibodies on the susceptibility and severity of various infectious diseases, including SARS-CoV-2 infection. In addition, we discuss the role of anti-IFN autoantibodies in the pathogenesis of autoimmune diseases that are characterized by recurrent infections.

## Introduction

In recent years, life-threatening diseases caused by anti-cytokine autoantibodies have received widespread attention, especially those in previously healthy adults. Patients usually demonstrate a unique infectious syndrome associated with high-titer neutralizing autoantibodies that target a specific cytokine. The autoantibodies may block the activity of the corresponding cytokine, which enhances susceptibility to one or more pathogenic bacteria. For example, granulocyte-macrophage colony-stimulating factor (GM-CSF) is required for surfactant degradation in the pulmonary alveoli and boosts innate immunity (1–3). Neutralizing anti-GM-CSF autoantibodies were discovered in patients with acquired pulmonary alveolar proteinosis (PAP), a respiratory disease in which surfactant protein accumulates in the lung (4, 5). Patients with acquired PAP are vulnerable to typical respiratory pathogens as well as opportunistic infections caused by pathogens such as *Nocardia* (6), *nontuberculous mycobacteria* (NTM) (7), *Histoplasma* (8), and *Cryptococcus* (9). Interleukin (IL)-17A, IL-17F, and IL-22 are considered to be important in mucosal immunity. They work together to produce proinflammatory cytokines that are important in neutrophil recruitment, granulopoiesis, and the generation of antimicrobial peptides such as S100 protein and defensins (10, 11). Chronic mucocutaneous candidiasis (CMC) has been observed in individuals with anti-IL-17A, anti-IL-17F, and anti-IL-22 autoantibodies, resulting from these autoantibodies blocking the Th-17 immunity (12). Furthermore, these autoantibodies and CMC are also linked to autoimmune polyendocrinopathy-candidiasis-ectodermal dystrophy syndrome (APECED) and thymoma (13). Other anti-cytokine autoantibodies, such as anti-IL-2 autoantibodies, have been found in human immunodeficiency virus (HIV)-infected patients (14); anti-G-CSF autoantibodies were identified in Felty’s syndrome (FS), in which neutropenia is associated with bacterial infections (15); and anti-IL-6 autoantibodies have been confirmed to be associated with severe bacterial infections, including *Escherichia coli*, Streptococcus intermedius, and Staphylococcus aureusinfections (14). In summary, anti-cytokine autoantibodies have been considered as an important etiology of immunodeficiency, though there are also many immunodeficiencies that do not rely on anti-cytokine autoantibodies, like GATA2 deficiency, one of the most prevalent causes of hereditary bone marrow failure. Its phenotype is distinguished by immunodeficiency, which results in invasive viral, mycobacterial, and fungal infections (16). In addition, there are other autoimmune manifestations observed with considerable frequency in patients with primary antibody deficiencies, including common variable immunodeficiency (CVID) and selective IgA deficiency (sIgAD), but also in patients with combined immunodeficiency disorders (CID) (17).

Interferons (IFNs) play a pivotal role in the immune system. They form the first line of defense in humans in response to pathogen invasion, especially against viral infection (18, 19). IFNs can be divided into three families, type I IFN (mostly IFN-α and IFN-β, several other subtypes also included), type II IFN (IFN-γ), and type III IFN (IFN-λ). All types of IFNs work through the JAK/STAT pathway where they bind receptors and sequentially active Janus family kinases (JAK and TYK) and signal transducer and activator of transcription (STAT), which leads to transcriptional activation of interferon stimulating genes (20).

Adult-onset immunodeficiency syndrome (AOID) differs from primary immunodeficiency disease (PID). Patients with AOID always have high titers of anti-IFN-γ autoantibodies, which is strongly associated with higher mortality (21–23). Since the coronavirus disease 2019 pneumonia (COVID-19) outbreak, it has been discovered in several studies that preexisting autoantibodies against type I IFNs (anti-IFN-I autoantibodies) are linked to the severity of COVID-19, which has aroused great interest in academic circles (24–26). Both AOID and COVID-19 are infectious diseases that usually lead to severe conditions in patients; therefore, the present study considers how anti-IFN autoantibodies are closely related to infection, particularly severe infectious diseases. This review summarizes the effects of anti-IFN autoantibodies on the susceptibility and severity of different infectious diseases. The role of anti-IFN autoantibodies in autoimmune diseases complicated with recurrent infection is also discussed.

## IFNs and Their Abs

IFN, first described by A. Isaacs and J. Lindenmann in 1957 (27), was thought to be an antiviral substance. The mechanism by which interferon acts against viruses is to stimulate the production of cytokines and the upregulation of antiviral effector proteins (28). Host cell death is an intrinsic cell defense mechanism during infection. Several studies have conclusively demonstrated that the necroptosis and pyroptotic cell death pathways are crucial for the release and action of inflammatory cytokines (29–31). Th1 cells, a separate subset of CD4^+^ Th cells, is also critical for protection against pathogens (32).

IFNs are the first cytokines discovered and provide the basis for understanding the structure, pathways, evolution, and functions of other cytokines and their receptors. They are also the first cytokines used in therapy (33). IFNs are used to treat autoimmune diseases including multiple sclerosis (34), tumors such as lymphoma (35), infectious diseases such as disseminated nocardiosis (36), and hepatitis C (37). Recently, it is even considered as a potential therapeutic target for COVID-19.

### Type I IFN

Type I IFNs, including IFN-α, IFN-β, IFN-ω, IFN-ϵ, IFN-κ, IFN-δ, and IFN-τ (33), are potent antiviral molecules, acting in both innate immunity (in particular *via* their secretion by plasmacytoid dendritic cells) and cell-intrinsic immunity (in most if not all cell types) (27). All these IFNs mentioned above bind to IFN-α receptor (IFNαR), which is composed of two subunits, IFNαR1 and IFNαR2. Once the type I IFNs bind to the receptor, the binding causes the phosphorylation of TYK2 and JAK1, which are associated with IFNαR1 and IFNαR2 separately; the phosphorylation of TYK2 and JAK1 subsequently leads to the phosphorylation of STAT1/STAT2. Activated STATs dimerize, translocate to the nucleus, and regulate type I IFN-inducible gene expression so as to stimulate the production of cytokines and the upregulation of antiviral effector proteins (28, 38). Duncan et al. reviewed monogenic lesions of type I IFN signaling pathways and informed our understanding of the type I IFN system within the concerted antiviral response (39).

Anti-IFN-I autoantibodies can be found in patients treated with IFN-a or IFN-β (40) and exist in almost all patients with autoimmune polyendocrine syndrome type-1 (APS-1) (41). They are also observed in patients with systemic lupus erythematosus (SLE) (42). Lately, they have been shown to affect life-threatening COVID-19 (24). Moreover, it is reported that inborn errors are more common in patients under the age of 60 years, whereas autoantibodies are more common in patients over the age of 70 years in COVID-19 (43).

### Type II IFN

Type II IFNs, also called IFN-γ, are generated mainly by natural killer (NK) cells, and NKT cells are one of the crucial cytokines for host defense against intracellular pathogens associated with antigen presentation, macrophage differentiation and activation, production of proinflammatory cytokines, cell death, tumor immunity, and autoimmunity (44). Cytokine-induced NK cell activation is regulated by IL-12. In the early stages of infection, phagocytes secrete IL-12 after binding with its receptor on NK cells, which results in activation of STAT4 and eventual production of IFN-γ. NK cell-mediated IFN-γ production promotes the activation of phagocytes, leads to increased secretion of IL-12 by phagocytes, and finally establishes a positive feedback loop (38, 45).

IFN-γ receptor (IFNγR) is presented in nearly every cell type, except for mature erythrocytes (46). The receptor is made up of IFNR1 and IFNR2 and belongs to the class II cytokine receptor family. Similar to type I IFNs, after binding to the IFNγR, IFN-γ activates the JAK-STAT pathway and mediates various biological responses (46, 47).

### Type III IFN

The type III IFN (refers to IFN-λ), first described in 2003, is the most recently recognized type of IFNs (48). IFN-λ is secreted by most cells but acts mainly on epithelial surfaces because of the restricted receptor expression (49).

**Figure 1:**
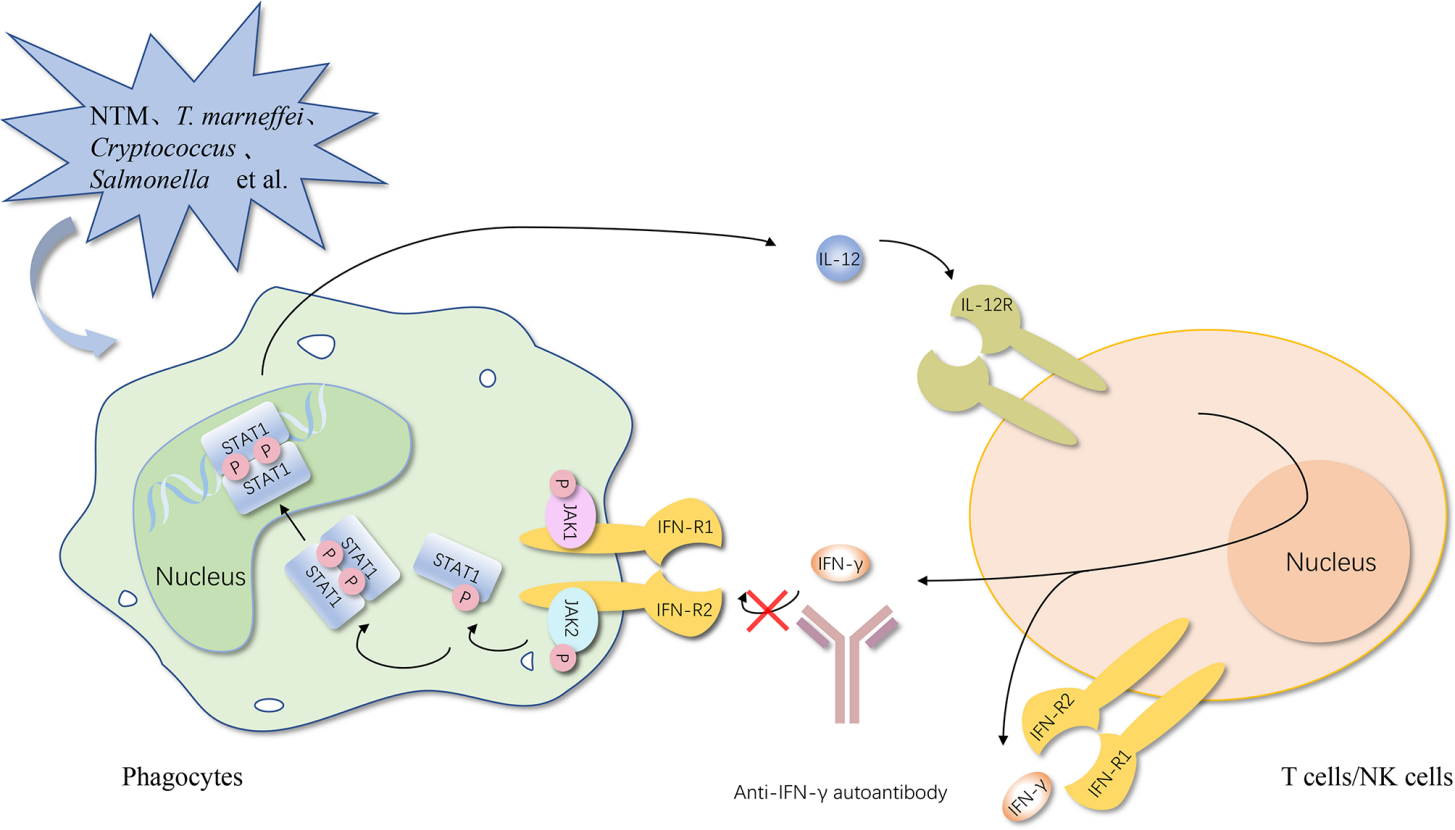
Anti-IFN-γ autoantibody increases the susceptibility to opportunistic pathogens *via* the IFN-γ/IL-12 axis. Once the opportunistic pathogens invade, activated phagocytes produce IL-12. After binding with its receptor on NK cells and NKT cells, IL-12 induces the secretion of IFN-γ. IFN-γ can bind to the IFN-γ receptor on phagocytes and promote the activation of phagocytes, thereby further increasing the production of anti-microbial proteins to control opportunistic pathogens and IL-12, establishing a positive feedback loop finally. Anti-IFN-γ autoantibody causes opportunistic infections by impairing the binding of type I IFNs to their receptor and the activation of the downstream responsive pathway. IFN, interferon; IFN-R, interferon-receptor; IL-12, interleukin-12; STAT, signal transducer and activator of transcription; P, phosphorylation; JAK, Janus kinase. The red cross represents the pathway blocked by the antibody.

**Figure 2:**
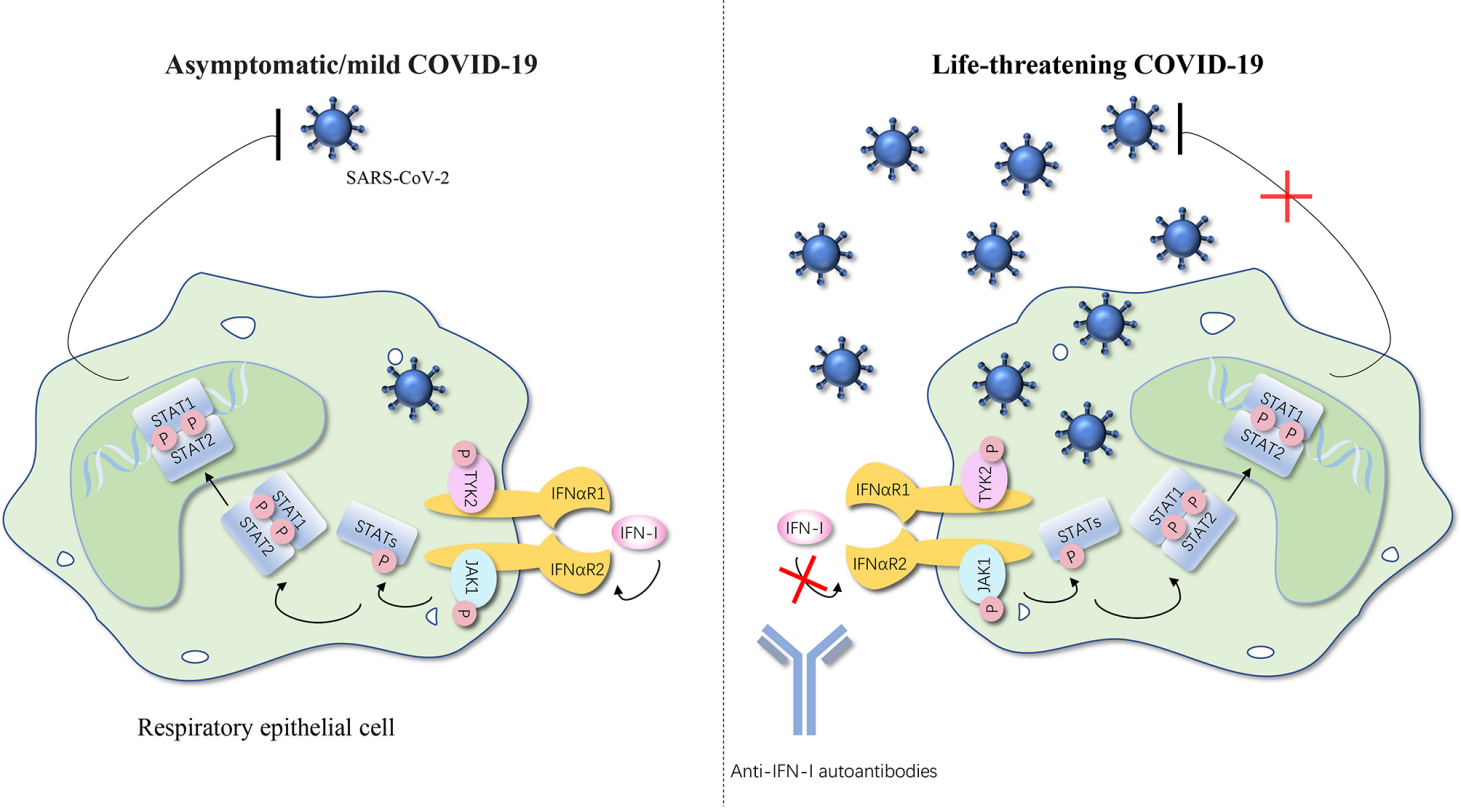
Anti-IFN-I autoantibodies underlie life-threatening COVID-19. IFN-I can bind to the IFNαR and cause the phosphorylation of TYK2 and JAK1, thereby further activating STATs. STATs translocate to the nucleus and regulate type I IFN–inducible gene expression to achieve the anti-SARS-CoV-2 activity. Autoantibodies against type I IFNs may underlie critical COVID-19 by blocking the binding of type I IFNs to their receptor and impairing type I IFN immunity. The number of SARS-CoV-2 represents the severity of the COVID-19. IFN, interferon; IFNαR, interferon-α receptor; STAT, signal transducer and activator of transcription; P, phosphorylation; JAK, Janus kinase; TYK, tyrosine kinase; SARS-CoV-2, severe acute respiratory syndrome coronavirus 2; COVID-19, coronavirus disease 2019 pneumonia. The red cross represents the pathway blocked by the autoantibodies.

In simple terms, no matter what type of interferons, anti-IFN autoantibodies with high titers in serum interrupt the activation of the downstream responsive pathway by blocking the combination between IFNs and their receptor and the consequence is increased infection rates (**Figures 1**, **2**) (50).

## Anti-IFN-γ Autoantibodies in AOID

Infection can be regarded as a normal event in anyone’s lifetime. However, if someone experiences chronic, recurrent, hard-to-control, or unusual serious infections of common pathogens compared with the normal population, then a potential deficiency of the immune defense can be considered.

Since 2004, some infectious diseases caused by opportunistic pathogens have been reported in adults without immunodeficiency (51–55). They were not infected with HIV but, similar to AIDS, showed clinical manifestations due to extremely low immunity. Browne et al. first came up with the term “adult-onset immunodeficiency syndrome” to describe this new type of disease because its major patient population is composed of adults (56). To date, a few studies regarding AOID have been conducted and published. The cumulative reports confirmed the critical role of anti-IFN-γ autoantibody in the pathogenesis of AOID (21, 57).

As we know, genetic deficiencies of the innate immune pathway lead to severe intracellular pathogen infections, which is known as Mendelian susceptibility to mycobacterial disease (MSMD), also called PID (58). PID are more common in children although both children and adults can be affected (58). However, for most reported AOID cases, infection occurred in adults aged around 50 years (18–78 years). Interestingly, almost all of the patients with AOID come from Asia, or live elsewhere but were born in Asia, which means that there is a distinct racial predisposition in the population (59). Accumulated reports strongly suggested that the AOID is highly associated with the expression of human leukocyte antigen (HLA). Chi et al. presented that HLA-DRB1*16:02 and HLA-DQB1*05:02 may account for the pathogenesis of NTM and *varicella-zoster virus* infection (57). HLA-DQB1*05:01 and HLA-DQB1*05:02 were also found in patients with a very high risk of critical AOID (60).

In addition, it is reported that both genetic factors and environmental exposure contribute to AOID. Moreover, autoimmunity caused by the anti-IFN-γ autoantibody plays an important role as well (21). Browne et al. showed that neutralizing anti-IFN-γ autoantibody was detectable in 88% of adults with multiple opportunistic infections in Asia, and up to 41 cytokine autoantibodies were detected in 97 opportunistic infection patients in Taiwan and Thailand. The results indicate that only anti-IFN-γ autoantibodies are closely associated with opportunistic infections in patients (56).

In patients with AOID, the clinical presentation, site of infection, and related pathogens may vary from one race to another. Thereinto, *Nontuberculous mycobacteria* (NTM) is the most common pathogenic microorganism (61). *Rapidly growing mycobacteria* (RGM), such as *Mycobacteria abscess*, are the most common NTM species isolated from patients in Thailand, China, and the Philippines while *Mycobacterium avium complex* (MAC) is mainly present in Japanese and non-Asian patients (59, 62). Lymph nodes are the most usual organ involved in patients with RGM infection, while bone and lung infections are the predominant apparatuses in patients with MAC infection (59). NTM are ubiquitous, low pathogenic environmental bacteria that widely exist in water, soil, and man-made environments (63, 64). The incidence of various infections caused by weakly virulent organisms increases year by year and has become more common than those caused by *Mycobacterium tuberculosis* (61). Disseminated NTM infections mainly occur in individuals with obvious immunodeficiencies, such as hairy cell leukemia patients, post-transplant patients, advanced HIV infection patients, individuals taking glucocorticoid or immunosuppressive therapies, and those with Mendelian defects in the IL-12/IFN-γ axis (65). Recently, an increasing number of disseminated NTM infections have been observed in HIV-negative individuals with no obvious immunosuppression, and the anti-IFN-γ autoantibody was identified as an important risk factor (64, 66, 67).

*Talaromyces marneffei* (*T. marneffei*) is another extremely important pathogen in AOID. *T. marneffei* infection in humans is supposed to be caused by inhalation of *T. marneffei* conidia in the environment (68). Upon entering the human body, it can replicate in yeast form in macrophages to cause infection, ranging from local infection in lungs and skin to disseminated systemic infection (69). The same as NTM infection, *T. marneffei* infection usually occurs in immunocompromised individuals with impaired cell-mediated immunity, including secondary immunodeficiency due to HIV infection, cancer, and immunosuppressive therapy. Among the patients with *T. marneffei* infection, the lungs and skin were the dominant organs affected (70). However, the patients with anti-IFN-γ autoantibody are more likely to have pleural effusion and multiple organ involvement (23). Chen et al. confirmed that patients who tested positive for anti-IFN-γ autoantibody usually have more frequently exhibited disseminated systemic infections with severe pleural effusion, have stronger inflammatory responses, are more likely to be infected by other intracellular pathogen, and have worse disease outcomes despite aggressive antimicrobial therapy (71).

In addition, other microorganisms including Salmonella species, Cryptococcus neoformans, Histoplasmosis capsulate, Burkholderia pseudomallei, Listeria, Varicella–zoster virus, Cytomegalovirus, and Epstein–Barr virus are also frequently identified as opportunistic pathogens in this patient group (56, 59, 72). Co-infection with at least 2 pathogens is more pinpoint to diagnose AOID.

## Abs Against Type I IFNs in Life-Threatening COVID-19

COVID-19, which is caused by severe acute respiratory syndrome coronavirus 2 (SARS-CoV-2), has become a serious public health emergency of international concern (73, 74). Up to September 1, 2021, it has infected over 200 million people around the world (https://coronavirus.jhu.edu/map.html). It is reported that most people infected with COVID-19 are asymptomatic or have just mild symptoms and about 20% may present with life-threatening complications, including multisystem organ failure, acute respiratory distress syndrome, and ultimately death (73, 75).

Risk factors for disease severity include older age, male sex, ethnicity, increased BMI, and pre-existing comorbidities such as chronic lung disease, cardiovascular disease, hypertension, diabetes, and cancer (76). Additionally, host genetic factors are also relevant to COVID-19 susceptibility and severity (77, 78). Pathogen recognition receptors of the innate immune system, e.g., toll-like receptors (TLRs), recognize pathogen-associated molecular patterns and activate IFN-related immune responses. They stimulate the production of proinflammatory cytokines *via* NF-κB activation and activate the interferon regulatory factors (IRFs), which induce type I IFN expression (79). IFNAR1 and IFNAR2 bind type I IFNs and are involved in signal transduction by activating the JAK-STAT pathway. Efficient IFN-I-mediated antiviral responses are critical for viral clearance. Zhang et al. reported that at least 3.5% of patients with life-threatening COVID-19 had known (AR IRF7 and IFNAR1 deficiencies or AD TLR3, TICAM1, TBK1, and IRF3 deficiencies) or new (AD UNC93B1, IRF7, IFNAR1, and IFNAR2 deficiencies) genetic defects at eight of the 13 candidate loci involved in the TLR3- and IRF7-dependent induction and amplification of type I IFNs (80). Chiara et al. found TLR7 deleterious variants in 2.1% of severely affected males and none of the asymptomatic participants (81). Autoimmune polyendocrine syndrome type 1 (APS-1), also known as APECED, is a monogenic inborn error of immunity typically caused by biallelic deleterious variants of the autoimmune regulator (*AIRE*) gene (82). Bastard et al. presented in their study that virtually all patients with APS-1 produce autoantibodies against type I IFNs, and 86% of them were hospitalized for COVID-19 pneumonia, including 68% who were admitted to an intensive care unit, 50% of whom required mechanical ventilation, and 18% of whom died (83). Moreover, other GWASs identified the SNP rs11385942 on chromosome 3, situated at 3p21.31 and around the genes SLC6A20, LZTFL1, CCR9, FYCO1, CXCR6, and XCR1, as a risk variant (84–86). Simply speaking, COVID-19 is characterized by excessive activation of both the innate and adaptive immune systems, giving rise to hyperinflammatory cytokine storms that mainly result in organ damage (87).

Anti-IFN-I autoantibodies account for life-threatening COVID-19 in at least 10.2% of patients. To be specific, it is detected in at least 2.6% of women and 12.5% of men with a life-threatening disease. As a control, it is undetectable in all 663 patients with asymptomatic or mild SARS-CoV-2 infection, and detectable in only 0.3% of 1227 healthy individuals (24). Furthermore, both *in vivo* and *in vitro*, these autoantibodies against type I IFNs showed their neutralizing activity, including the ability to block SARS-CoV-2 infection (24). Collectively, these data provide compelling evidence that neutralizing anti-IFN-I autoantibodies are important to the elimination of SARS-CoV-2 infection and can even explain the severity of the disease (24).

Neutralizing anti-IFN-I autoantibodies may impair the binding of type I IFNs to IFNαR and the activation of the downstream pathway, block the antiviral effect of type I IFNs, and result in life-threatening COVID-19. Bastard et al. also constructed a two-step model to explain the pathogenesis of life-threatening COVID-19 with insufficient type I IFN immunity during the first few days of infection resulting in underlying viral growth and dissemination and leading to the unleashing of damaging secondary, excessive pulmonary, and systemic inflammation (24–26).

## Therapy Strategies of Diseases Associated With Anti-IFN Abs

Standardized antimicrobial therapy is the most important treatment but it is still difficult to cure just by using antibiotics in AOID patients with anti-IFN-γ autoantibody (88). Decreasing the titer of anti-IFN-γ autoantibody is considered the key to curing patients. Likewise, as a substitution to ectogenous type I IFNs, treatments including plasmapheresis to deplete monoclonal antibodies, some specific interventions to inhibit the production of reactive B cells, and plasmablast depletion were confirmed to be beneficial in COVID-19 patients with autoantibodies against type I IFNs (24, 83, 89)

### Cyclophosphamide

Cyclophosphamide is a nitrogen mustard analog, which exerts its effects through inhibition of DNA replication. The drug acts by activating capable of preventing protein synthesis through DNA and RNA crosslinking and eventually inhibits cell division (90, 91). Cyclophosphamide has been used to treat several neoplastic diseases such as lymphomas, leukemia, ovary, and breast cancer, and is also identified as an immunosuppressant (91). Chetchotisakd et al. reported the use of pulse intravenous cyclophosphamide in 7 patients who had refractory, progressive *Mycobacterium abscessus* infection. Finally, 5 patients had favorable outcomes without hospitalization while 2 patients were hospitalized due to relapse. Even more to the point, the anti-IFN-γ autoantibody titers among all the 7 patients significantly decreased during treatment, and the degrees of reduction among satisfactory prognosis vs. poor prognosis were significantly different (92).

### Rituximab

Rituximab is a monoclonal antibody binding to the CD20 antigen and used to treat types of cancer and certain autoimmune diseases (93). Browne et al. used rituximab in 4 previously healthy women with a high-titer anti-IFN-γ autoantibody and disseminated NTM who had progressive disease despite aggressive antimicrobial therapy. After receiving differing amounts of rituximab, all patients achieved clinical remission along with a reduction in antibody titer and relief of inhibition in the IFN-γ pathway (94). Carlos et al. presented in the literature the first case of clinical remission in a patient who had prolonged COVID-19 symptoms by using monoclonal neutralizing antibodies (95).

### Daratumumab

Daratumumab is an IgGκ monoclonal antibody targeting CD38, which is highly expressed in plasma cells, plasmablasts, B cells in early maturation stages, and myeloma cells (96). Prior studies have shown that CD38 is an effective treatment for multiple myeloma patients with a striking reduction of RF, ANCA, and ANA titer (97). Sebastian et al. described the first use of daratumumab in a patient with no prior history of immunodeficiency disease who had a high titer of neutralizing anti-IFN-γ autoantibody. The patient’s disease was not under control despite receiving various antibiotics and multiple cycles of rituximab and bortezomib. However, treatment with daratumumab eventually resulted in clinical and radiographic improvement (98).

### Exogenous IFN

Exogenous recombinant IFN binds to circulating antibodies, resulting in inhibition of their pathogenicity. Here, timing is of the essence. In COVID-19, early application of IFN was beneficial to decrease viral load and improved the prognosis of disease, whereas delayed IFN treatment was of no benefit compared with the placebo group (99). Concerning AOID, Harada et al. presented a case of a 65-year-old Japanese man who was infected with NTM. In this case, the antibiotics treatment did not function well enough and exogenous subcutaneous injection of IFN-γ therapy had a good response (100).

### Plasma Exchange

Therapeutic plasma exchange is a plasma purification treatment capable of eliminating large molecular weight substances from blood (101). It is the fastest way to remove circulating autoantibodies. Nicolas et al. reported four patients with life-threatening COVID-19 who were treated with plasma exchange as a rescue therapy (89).

### Other Treatments

Transfusion of convalescent plasma to infected patients has emerged as an effective therapy for severe COVID-19. It attenuates the severe inflammatory response by providing cytokines and other factors regulating the immune response against SARS-CoV-2 (75).

In conclusion, these reports suggest that patients with anti-IFNs autoantibodies always have a chronic, persistent infection, and we should pay attention to long-term treatment. Cyclophosphamide, autoantibody depletion with plasma exchange, B-cell depletion with anti-CD20 antibody, and supplementation of recombinant IFN have been successfully used as adjuvant therapies combined with antimicrobial therapy in a small percentage of patients. Among patients treated with antibiotics alone, antibiotics and rituximab, and antibiotics and cyclophosphamide, the median time to infection clearance was 3, 4, and 5 years, respectively (59).

## Prospect of Anti-IFNs Autoantibodies in Autoimmune Diseases

In autoimmune diseases, autoantibodies against one cytokine or more cytokines have been reported, but their spectrum and clinical effect remain largely unknown (38, 42). In different circumstances, they may be beneficial or harmful, depending not only on the activity of the autoantibodies themselves but also on the intrinsic role of the target cytokines. It is noteworthy that autoantibodies against type I and type II IFN in serum were detected in up to 21.6% of patients with SLE (42). Their effect on IFN gene expression characteristics and pathogenesis of SLE is unknown, but they influence IFN signaling, disease activity, and response to biologic therapy (102). On the one hand, their existence may help to classify patients with SLE or other autoimmune diseases currently diagnosed with similar pathological features. Gupta et al. used the luciferase immunoprecipitation system method to detect and quantify 24 different anti-cytokine autoantibodies and evaluated a total of 498 patients diagnosed with SLE, primary Sjogren’s syndrome, or rheumatoid arthritis. Eventually, the increased SLEDAI score and abnormal laboratory tests in SLE patients were almost entirely attributable to the presence of anti-IFN-γ autoantibody (42). On the other hand, despite the continuous progress in the treatment of SLE in recent years, infection is still the primary cause of morbidity and death of SLE patients (103). We noted that some patients with SLE often present with recurrent and refractory infections clinically, which are still difficult to control and prone to recurrence despite being treated with antibiotics aggressively. Among them, cytomegalovirus, *Epstein–Barr virus*, and *varicella-zoster virus* are the most common pathogenic agent, and *Klebsiella pneumoniae*, *Acinetobacter baumannii*, and *Cryptococcus neoformans* are not rare. These pathogens are also involved in AOID.

Thus, we believe that the level of neutralizing anti-IFN autoantibodies in patients with SLE and other autoimmune diseases complicated with repeated infection deserves further investigation. It may have important suggestive significance for the prognosis and outcome of the disease, and may also have important value in guiding the treatment of this kind of disease.

## Conclusion

To date, cumulative reports and studies suggest that anti-IFN-I autoantibodies play an important role in life-threatening COVID-19. Meanwhile, anti-IFN-γ autoantibodies are crucial to AOID. Anti-cytokine autoantibodies can have severe consequences as well as highly varied manifestations. It is necessary to detect serum anti-IFN autoantibody levels for patients with clinical manifestations of multiple opportunistic pathogen infections or refractory infections. At present, no standard treatment guidelines have been established for anti-IFN autoantibody-related infections, and control of infection combined with immunotherapy is a feasible method. In autoimmune diseases, the growing importance of cytokines and cytokine autoantibodies is starting to emerge, especially since we are now in an era of targeted therapy using biological preparations. A better understanding of the resistant properties and biological functions of cytokine autoantibodies in autoimmune disease patients may help to design future treatment. Further research to study key questions regarding the pathogenicity of anti-IFN autoantibodies in autoimmune disease patients infected with opportunistic pathogens will become feasible.

## Author Contributions

LC constructed the figures and wrote the manuscript. CY designed the review’s structure and revised the manuscript. XC designed the figures and revised the manuscript. All authors contributed to the review and approved the submitted version.

## Funding

This work was supported by the National Natural Science Foundation of China (81801596).

## Conflict of Interest

The authors declare that the research was conducted in the absence of any commercial or financial relationships that could be construed as a potential conflict of interest.

## Publisher’s Note

All claims expressed in this article are solely those of the authors and do not necessarily represent those of their affiliated organizations, or those of the publisher, the editors and the reviewers. Any product that may be evaluated in this article, or claim that may be made by its manufacturer, is not guaranteed or endorsed by the publisher.
